# Therapy Effects of Advanced Hypopharyngeal and Laryngeal Squamous Cell Carcinoma: Evaluated using Dual-Energy CT Quantitative Parameters

**DOI:** 10.1038/s41598-018-27341-0

**Published:** 2018-06-13

**Authors:** Liang Yang, Dehong Luo, Junlin Yi, Lin Li, Yanfeng Zhao, Meng Lin, Wei Guo, Lei Hu, Chunwu Zhou

**Affiliations:** 1 Department of Diagnostic Radiology, Beijing Chaoyang Hospital, Capital Medical University, Beijing, 100020 China; 2 Department of Diagnostic Radiology, National Cancer Center/Cancer Hospital, Chinese Academy of Medical Sciences and Peking Union Medical College, Beijing, 100021 China; 3 Department of Radiotherapy, National Cancer Center/Cancer Hospital, Chinese Academy of Medical Sciences and Peking Union Medical College, Beijing, 100021 China

## Abstract

The accurate evaluation of the therapeutic effects of advanced laryngeal and hypopharyngeal squamous cell carcinoma (LHSCC) remains challenging. In this study, we determined the value of quantitative parameters derived from dual-energy computed tomography (DECT) for predicting the therapeutic effects of advanced LHSCC and to provide valuable evidence for early judgement of the tumour’s response to therapy in clinical practice. We prospectively analysed 41 patients with pathologically confirmed LHSCC. All patients received a DECT scan before therapy. Nineteen of 41 patients showed complete remission (CR), and 22 showed non-complete remission (NCR). The mean of the slope of spectral Hounsfield unit curve (λ_HU_), standardized iodine concentration and effective atomic number in the CR group were significantly lower than the NCR group (*P* < 0.05). There were no significant differences for T stage, treatment modality and standardized water concentration between two groups (*P* > 0.05). The best predictor of CR effect was λ_HU_. The 2-year cumulative recurrence rate of patients with higher λ_HU_ values was significantly higher than that of patients with lower λ_HU_ values (*P* < 0.05), while the 2-year survival rate of those patients was not significantly different (*P* > 0.05). DECT could easily identify CR patients and potentially help to choose the appropriate treatment regimen for advanced LHSCC.

## Introduction

Laryngeal and hypopharyngeal cancer are the most common cancers in the head and neck, most of which are squamous cell carcinoma (over 90%); other types are rare^1,2^. Approximately 40% of laryngeal cancer patients and 60% of hypopharyngeal cancer patients are diagnosed at clinical stage III or IV, known as advanced laryngeal and hypopharyngeal squamous cell carcinoma (LHSCC). The comprehensive surgical management is the standard option for LHSCC; however, in resectable patients, when the anticipated functional outcome and/or the prognosis is so poor that mutilating surgery is not justified^3–5^. On the other hand, combined concomitant chemoradiation is also the standard treatment in nonresectable patients^6^. In 1991, the Laryngeal Cancer Group of American Veterans discovered the laryngeal function-sparing effects of inducing chemotherapy and radiotherapy^7^. In 2003, the US RTOG 91–11 study confirmed that laryngeal function sparing rates of concurrent radiotherapy and chemotherapy (CRC), radiotherapy (RT), induction chemotherapy + radiotherapy (IC + RT) were 84%, 66%, and 71%, respectively^8^. In 2006, Bonner *et al*. published a remarkable phase III clinical trial that showed that cetuximab, a representative of the epidermal growth factor receptor inhibitors (EGFRI), combined with radiotherapy, is a therapeutic option^9^. Currently, non-surgical treatments, including CRC, IC + RT and RT combined with EGFRI, have been the available options for preserving organ function in laryngeal and hypopharyngeal cancer patients^10–12^. However, some patients are not sensitive to radiotherapy and need salvage surgery. In those patients, salvage surgery after radical radiotherapy would increase the risk of operative complications. If those RT non-sensitive patients could be identified and have salvage surgery performed, the adverse effects of RT could be avoided.

Currently, there is no optimal imaging method available to evaluate the radio-sensitivity of laryngeal and hypopharyngeal cancer. Studies have shown that dynamic contrast-enhanced CT, MRI functional imaging and PET-CT have a certain ability to evaluate the treatment effects of LHSCC, but drawbacks such as high cost, long scanning time and poor resolution of small lesions limit their clinical application. Dual-energy CT (DECT) could result in quantitative and qualitative analyses through energy spectrum parameters of the tissue, creating a new field of CT imaging^13–19^. Thus, the purpose of this study was to prospectively compare the performance of quantitative parameters derived from dual-energy CT in the early prediction of therapeutic responses of advanced LHSCC patients.

## Results

### Patients’ characteristics

Of the 48 patients enrolled, 7 patients were excluded from this study due to (a) no measurable lesions in 2 cases, (b) withdrawal of consent in 2 cases, and (c) lack of completion of the full course of therapy in 3 cases. Thus, 41 patients (mean age, 56.26 years; range, 38–77 years) with advanced LHSCC composed our study group. All patients enrolled had clinical stage III/IV, M0 disease. Patients presenting with technically resectable disease were offered non-surgical therapy in an attempt to preserve speech and/or swallowing function. In the CR group (n = 19), the average age was 57.37 ± 10.61 years. The cancers included laryngeal squamous cell carcinoma, 3 cases (15.79%); hypopharyngeal squamous cell carcinoma, 16 cases (84.21%); clinical stage III, 2 cases (10.53%); clinical stage IV, 17 cases (89.47%). In the NCR group (n = 22), the average age was 54.91 ± 7.54 years; The cancers included laryngeal squamous cell carcinoma, 3 cases (13.64%); hypopharyngeal squamous cell carcinoma, 19 cases (86.36%); clinical stage III, 2 cases (9.09%); clinical stage IV, 20 cases (90.91%).

### The difference in the constituent ratio of the T stage and treatment modality

There was no significant difference in the constituent ratio of the T stage and treatment modality between the two groups (*P* > 0.05) (shown in Table 1).

**Table 1:** The difference of constituent ratio of T stage, treatment modality.

		CR group (n = 19)	NCR group (n = 22)	*P*
T stage (%)	T1	2 (10.53%)	1 (4.55%)	0.78
	T2	4 (21.05%)	7 (31.82%)	
	T3	3 (15.79%)	4 (18.18%)	
	T4	10 (52.63%)	10 (45.45%)	
Treatment modality (%)	RT	4 (47.06%)	5 (22.73%)	0.35
	CRT	4 (21.05%)	8 (36.36%)	
	IC + RT	2 (10.53%)	3 (13.64%)	
	IC + CRT	7 (36.84%)	4 (18.18%)	
	RT + CTX	2 (10.53%)	0	
	IC + CRT + CTX	0	2 (9.09%)	

### Differences in Quantitative Parameters of DECT between Different Therapeutic Effect Groups

**Figure 1:**
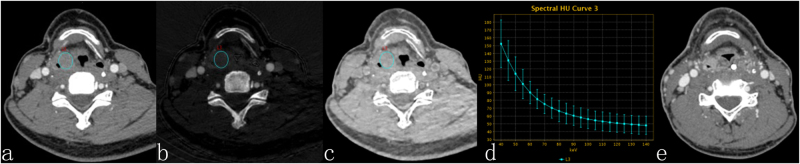
44Y, M, hypopharyngeal squamous cell carcinoma. Contrast-enhanced GSI images before RT (**a**,**b**,**c**) and three months after therapy. (**a**) The 70-keV monochromatic image shows right pyriform sinus cancer. (**b**) The iodine-based material-decomposition image shows that the IC-L of ROI is 17.27·100 μg/cm^3^ (sIC = 0.29). (**c**) The water-based material-decomposition image shows that the WC-L of ROI is 1038.57 mg/cm^3^ (sWC = 1.01). (**d**) Spectral HU curve of ROI; λ_HU_ was 2.45. (**e**) The 70-keV monochromatic image shows the residual tumour in the right pyriform sinus, and the response assessment is NCR.

**Figure 2:**
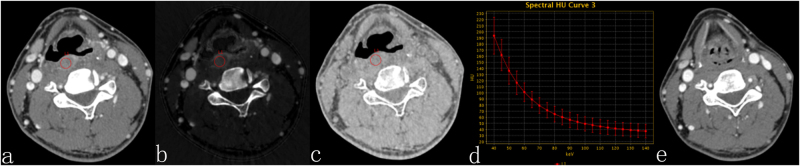
54Y, M, hypopharyngeal squamous cell carcinoma. Contrast-enhanced GSI images before CRT (**a**,**b**,**c**) and three months after therapy. (**a**) The 70-keV monochromatic image shows posterior pharyngeal wall cancer. (**b**) The iodine-based material-decomposition image shows that the IC-L of the ROI is 15.11·100 μg/cm^3^ (sIC = 0.18). (**c**) The water-based material-decomposition image shows that the WC-L of the ROI is 1033.43 mg/cm^3^ (sWC = 1.00). (**d**) Spectral HU curve of ROI; λ_HU_ was 1.92. (**e**) The 70-keV monochromatic image shows that the mucous membrane of the posterior pharyngeal wall is smooth, and the response assessment is CR.

The results of the statistical analysis of the quantitative GSI parameters indicated that the sIC, λ_HU,_ and Z_eff_ of the CR group were significantly lower than that of the NCR group, while there was no significant difference in the sWC between the two groups (Table 2). The mean sIC (*P* < 0.00), λ_HU_ (*P* < 0.00)_,_ and Z_eff_ (*P* < 0.02) for the CR group were (20.51 ± 5.092 [standard deviation]) × 10^−2^, 1.96 ± 0.52 and 8.51 ± 0.20 VS (30.41 ± 9.61) × 10^−2^, 2.72 ± 0.64, 8.71 ± 0.29 for the NCR group. In addition, the mean sWC (*P* > 0.05) for the CR group was (1005.59 ± 6.67) × 10^−3^ VS (1003.98 ± 11.41) × 10^−3^ for the NCR group (Table 2, Figs 1 and 2).

**Table 2:** The differences in quantitative parameters of DECT between CR and NCR groups.

	CR group ($\bar{x}\pm s$)	NCR group ($\bar{x}\pm s$)	*t*	*P*
sIC	(20.51 ± 5.09) × 10^−2^	(30.41 ± 9.61) × 10^−2^	4.59	0.00*
sWC	(1005.59 ± 6.67) × 10^−3^	(1003.98 ± 11.41) × 10^−3^	0.54	0.59
λ_HU_	1.96 ± 0.52	2.72 ± 0.64	−4.13	0.00*
Z_eff_	8.51 ± 0.20	8.71 ± 0.29	−2.44	0.02*

*Statistically significant.

### Quantitative Parameters of DECT as Predictors of Therapeutic Response

An independent sample t-test showed that the potential predictors of response to therapy were sIC, λ_HU_ and Z_eff_. A logistic regression model was constructed to predict the response using those quantitative parameters of DECT. The result indicated that λ_HU_ was the best predictor, and could discriminate CR from NCR. The adjusted OR for λ_HU_ was 13.91 (Table 3), that is, for every unit increase in λ_HU_, the odds of the CR response to therapy would be increased by a factor of 13.91 times

**Table 3:** The binary logistic regression of λ_HU_.

	$\hat{\beta }$	S.E.	Wald *χ*^2^	OR	*P*
λ_HU_	2.63	0.93	8.07	13.91	0.004*

*Statistically significant.

### Quantitative Parameters of DECT for Differentiating Therapeutic Response

**Figure 3:**
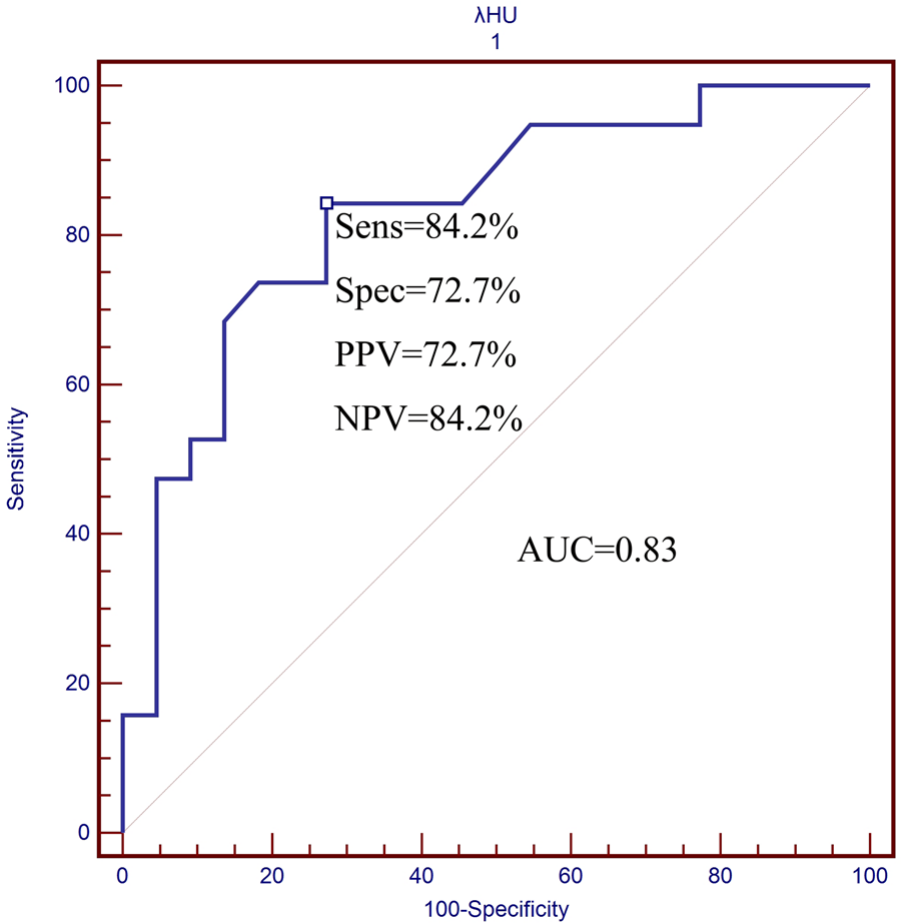
ROC curves. The graphs show the slope of the spectral HU curve for differentiating the CR and NCR therapeutic effect. The best threshold value for predictive probability of λ_HU_ for the differentiating CR from NCR was 2.71. AUC = area under the curve.

The final logistic regression model using λ_HU_ for predicting the CR effect to therapy had an area under the ROC curve of 0.83. With λ_HU_ ≤ 2.37 as the optimal diagnosis threshold in the prediction of CR, sensitivity, specificity, Youden’s index, positive likelihood ratio(+LR), negative likelihood ratio (−LR), positive predictive value(PPV) and negative predictive value (NPV) were 84.21% (95% CI: 60.4%, 96.4%), 72.73% (95% CI: 49.8%, 89.2%), 0.57, 3.09, 0.22, 72.7% and 84.2%, respectively (Fig. 3).

### Two-year Results of Patients with Different λ_HU_ Values in LHSCC

**Figure 4:**
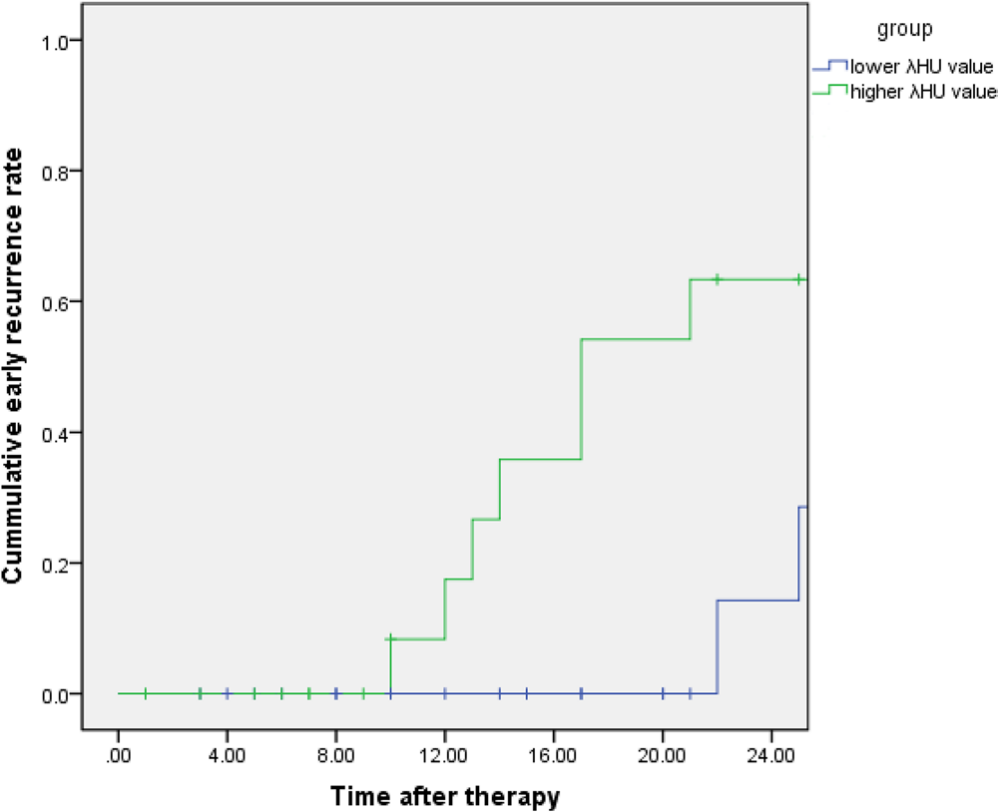
Two-year cumulative early recurrence rate in the lower and higher λ_HU_ group. The graphs show that the two-year cumulative early recurrence rate in the higher λ_HU_ group is significantly higher than that of the lower λ_HU_ group.

By the end of November 2017, a total of 25 patients were followed-up for more than two years. The median follow-up period was 20 months (range, 3–34 months), and the median age was 56 years (range, 38–77 years). According to different values of λ_HU_ (λ_HU_ ≤ 2.37 or λ_HU_ > 2.37), patients were divided into a lower λ_HU_ group (12 cases) and a higher λ_HU_ group (13 cases). During the follow-up period, there were two deaths (died of local recurrence) in the higher λ_HU_ group and three deaths (two died of local recurrence, one died of distant metastasis) in the lower λ_HU_ group. The overall 2-year early survival rate was 4.88% and 7.32% respectively, and there was no significant difference between groups (*P* > 0.05). Five and 8 patients with initial therapy subsequently experienced a local recurrence in the lower and higher λ_HU_ group, respectively. The 2-year cumulative early recurrence rate was 12.20% in the lower λ_HU_ group and 19.51% in the higher λ_HU_ group, and the difference was statistically significant (*P* < 0.05) (Fig. 4).

## Discussion

The efficacy of radiotherapy for LHSCC is certain, while chemotherapy shows some limited effects. Some studies suggest that chemotherapy plays a limited role in shrinking tumour volume and increasing long-term survival rate^20,21^. Due to the difficulty in quantifying the exact therapeutic effects of chemotherapy, this study takes radiotherapy as the primary research subject, and all participants underwent the standard course of radiotherapy.

The main factors affecting the RT efficacy in advanced LHSCC are radiosensitivity, clinical stage, degree of differentiation, tumour volume, haemoglobin level and treatment methods^22,23^, among which radiosensitivity is the most important factor. The radiosensitivity of tumour cells is related to the blood supply and oxygen content of the tumour^24^. Due to tumour vascular maldevelopment and microcirculation disorder, solid tumours usually contain a certain proportion of hypoxic cells, which have 3 times lower of radiosensitivity than fully oxygenated tumour cells^25^. The tissue oxygen level is directly related to the blood supply.

T staging is one of the factors affecting the treatment effect and may have an impact on the energy spectrum parameters. The comparison of T stage showed there was no significant difference (*P* > 0.05) between the NCR and CR group in this study, which demonstrated that the distribution of T stage was balanced in the two groups and excluded the impact of T staging on spectrum parameters. Although there was no uniform treatment in the study, there was no significant difference in treatment constituents between the two groups (*P* > 0.05). Therefore, the impact of treatments on the CR group and NCR group spectrum parameters could be considered balanced.

The λ_HU_ reflects the attenuation in HU values of tissue or materials across the 40~140 keV range. The linear attenuation coefficient of substances declines with increased keV, but each material has a different decline rate. In other words, the λ_HU_ can reflect the character of energy decay and is determined by the physical and chemical properties of the material itself, which are exploited to enhance the contrast between different tissues at any selected keV^26,27^. Meanwhile, DECT can calculate the Z_eff_ of any voxel by measuring the linear coefficient at 2 different tube potentials, which can help identify different tissue types^28,29^. A previous study showed that each tissue had a characteristic λ_HU_ and Z_eff,_ which had high sensitivity and specificity for differentiating benign and malignant neck pathologic findings^30^. In our study, the λ_HU_ between the CR and NCR group were significantly different, which indicated that the two groups have different organizational natures. However, the difference in the Z_eff_ between the two groups was not as significant as the λ_HU,_ which could be due to the small sample size of this study.

In spectrum imaging, any structure or organization could be shown by a combination of two base materials to produce the same attenuation effect. Iodine is the main component of the CT contrast agent, and water is the most common substance in the human body. Therefore, water - iodine is the most commonly used base material combination in enhanced scan spectrum imaging^31,32^. DECT can perform accurate quantitative analysis of the iodine concentration in the tissue^33,34^, and thus the spectrum analysis results indicate the tumour blood supply, which is the theoretical basis of DECT to assess tumour treatment efficacy by its material analysis function.

In this study, the CR group had sIC values lower than the NCR group, and the results were statistically significant difference. The sIC is influenced by tumour vascular permeability and microcirculation perfusion. The NCR group had higher vascular permeability, an increased number of incomplete vascular endothelial cells, and larger gaps between adjacent endothelial cells, causing more contrast agent to leak out of the blood vessel in the enhancing scan.

The ROC curve illustrates both the sensitivity and specificity of certain diagnostic methods and accurately reflects their relationship, which represents a comprehensive indicator of the test’s accuracy^35^. In this study, the AUC value of sIC for predicting the therapeutic effects of advanced LHSCC reaching to CR was over 0.7, thus indicating a moderate diagnostic value of sIC and λ_HU_^36,37^. We used λ_HU_ ≤ 2.37 as a threshold to predict that CR had a higher sensitivity (84.21%) after treatment. These studies showed that no matter what treatment option was chosen, advanced LHSCC patients with the higher λ_HU_ value were always more likely to have NCR. The 2-year follow-up also confirmed that patients with a higher λ_HU_ values were more prone to have early local recurrence (41.67% VS 61.54%, *P* < 0.05). The study revealed that patients with higher λ_HU_ values had a significantly lower risk of progression and local recurrence. Although the 2-year survival rates of the lower λ_HU_ group were lower than that of the higher λ_HU_ group, there was no significant difference (16.77% VS 23.08%, *P* > 0.05), possibly because of the short follow-up times in this study. A previous study suggested that after treatment, the advanced LHSCC patients with NCR had higher localized control failure rates than the CR patients^38^, and other studies showed that the main factors affecting the prognosis of advanced LHSCC patients were local recurrence and metastasis^39^. Once local recurrence occurred, the overall survival time decreased significantly to a median of 5–26 months^40–42^.

There were several limitations in this study. First, it was conducted in a single centre with a relatively small number of subjects and short follow-up times, which might result in limitations of the prediction model used in our study. Thus, larger prospective studies would be needed to determine whether this predictive model can be applied to other tumour subsets or histological findings. With more clinical data available, the correlation between tumour initial DECT quantitative parameters and long-term therapeutic effects (e.g., length of local control, disease-free intervals, etc.) could be established, which would provide valuable information for patients to choose between surgery and an organ-preservation protocol in clinical settings.

In conclusion, this study demonstrated that elevated DECT quantitative parameters in pretherapy patients with local advanced LHSCC were statistically correlated to a therapeutic response to a trial of radiotherapy with/without chemotherapy. The results suggested that DECT could be a potential method for evaluating the therapeutic response of advanced LHSCC. The study also showed that DECT quantitative parameters might be useful in clinical practice as a tool to help stratify patients into appropriate treatment arms, reduce the time to definitive therapy, and limit or eliminate unnecessary therapy.

## Materials and Methods

### Inclusion and exclusion criteria

This prospective single-institution study was approved by the institutional review board of the Cancer Institute & Hospital, Chinese Academy of Medical Sciences. Informed consent was obtained from all participants. This study was carried out in accordance with the Declaration of Helsinki.

From January 2014 to December 2016, a total of 48 patients with previously untreated, advanced LHSCC were enrolled in the study. Each patient was evaluated by a multidisciplinary physician team including a surgeon, medical oncologist, and radiation oncologist before providing signed study consent. Patients were deemed eligible if they presented with an unresectable tumour or if a planned surgery would have a significant adverse impact on long-term speech and/or swallowing function.

Inclusion criteria were as follows: (a) pathologically confirmed primary LHSCC, (b) age >18 years, (c) clinical stage III-IV, with an expected survival time over 12 months, (d) more than one measurable lesion that could be identified by CT or MRI, (e) Karnofsky Performance Status ≥70, and normal haematopoietic, hepatic, and renal functions, (f) no evidence of early distant metastasis, and (g) no contraindications of radiotherapy.

### CT Examination

All patients were confirmed by histological biopsy or pathological examinations and underwent DECT (Discovery CT750 HD, GE Healthcare) with gemstone spectral imaging (GSI) mode before treatments. The scanning parameters were: GSI-17 protocol (manufacturer number), helical mode, axial plane with coverage from mid orbits to the clavicular heads, collimator of 20 mm, slice thickness of 1.25 mm, slice interval of 0.8 mm, pitch of 0.984, tube current of 550 mA, tube voltage fast switching between 80 kVp and 140 kVp with cycle of 0.5 ms, SFOV of large body. All patients were intravenously injected with contrast media (Ultravist 300; Bayer Pharma AG, Leverkusen, Germany) by using a power injector with a rate of 2.5 ml/s, and volume of 1.5 ml/kg (85–100 ml). The scan acquisition was started with a delay of 30 s after start of injection.

### Image analysis

The original data acquired were reconstructed into monochromatic images. The reconstructed images were then sent to a post-processing workstation (Advantage Workstation 4.6, GE Healthcare, Milwaukee, WI). In the axial image, a radiologist with 10 years’ experience in CT diagnosis of head and neck tumours selected the maximum level of the lesion and sketched the region of interest (ROI) manually. The ROI was drawn to be as large as possible to include the whole lesion, with care to exclude peripheral fat, blood vessels, necrosis, and calcifications. The quantitative parameters were measured, including the effective atomic number (Z_eff_), the iodine concentration of the lesion (IC-L), the water concentration of the lesion (WC-L), the iodine concentration of the right carotid sinus (IC-C) and the water concentration of the right carotid sinus (WC-C). The IC-L and WC-L were standardized to values in the right carotid sinus (IC and WC) to obtain a standardized IC (sIC) and a standardized WC (sWC): sIC = IC-L/IC-C) and sWC = WC-L/WC-C. The slope of the spectral HU curve (λ_HU_) was calculated as the difference between the CT value at 40 keV and that at 90 keV divided by the energy difference (50 keV): λ_HU_ = (CT_40keV_ − CT_90keV_)/50.

### The classification criteria of LHSCC

The TNM classification criteria of the tumour was based on the classification criteria of the International Union of Counter Cancer(UICC) and the American Joint Committee on Cancer (AJCC) (2010)^43^.

### Treatment regimen

Treatment options were determined together by one radiation oncologist and one medical oncologist. Radiotherapy (RT) was delivered at 2.12 Gy per day, 5 days per week, to a total dose of 69.96 Gy for the primary tumour. Uninvolved nodal chains received 50 Gy, whereas chains harbouring grossly involved nodes received 60 Gy. RT treatment plan generation used intensity-modulated radiation therapy (IMRT) planning techniques, and all patients were irradiated by a 6 MV external beam. Concurrent chemotherapy with a course of gemcitabine was administered intravenously over 30 minutes once a week, 1–2 hours before radiotherapy, for 7 consecutive weeks, at a dose of 30 mg/m^2^. EGFRI was used as cetuximab (CTX). One week before radiation, the CTX loading dose of 400 mg/m^2^ was infused over 2 h. This was followed by weekly infusions at 250 mg/m^2^. Induction chemotherapy (IC) included 2 cycles: one cycle contained cisplatin (100 mg/m^2^) on day 1, followed by 5-fluorouracil (1000 mg/m^2^) treatment daily for 5 days.

### Response assessment

According to general examination methods (contrast-enhanced MR and/or CT, panendoscopy), the tumour responses were evaluated by a radiation oncologist and a radiologist three months after the completion of treatment based on the solid tumour’s effect evaluation criterion (RECIST 1.1). According to the therapeutic effect, the patients were divided into a complete remission (CR) and non-complete remission (NCR) group [including the patients with partial remission (PR), stable disease (SD) and progressive disease (PD)].

### Statistical analysis

The database was created with Microsoft Excel, and statistical analysis was performed with SPSS 19.0 (SPSS Inc., Chicago, IL) statistical software package. Quantitative data with a Gaussian distribution were presented as the mean ± standard deviation (*X* ± *S*), while quantitative data with a non-normal distribution were presented as (median, inter-quantile range) or *M* (*P*_25_-*P*_75_). The normality and homogeneity of variance of all measurement data were analysed, and if the data had a normal distribution and homogeneity of variance, an independent sample t-test was used. If the data did not have a normal distribution or homogeneity of variance, a Wilcoxon rank sum test was used. The proportion of T stage, clinical stage and treatment modality were compared by a Chi-square (*χ*^2^) test. Logistic regression models with the generalized estimating equations method were used to evaluate the predictive values. The adjusted odds ratio (OR) and its confidence interval were obtained from the final model as a measure of the association between the predictor and response. Receiver operating characteristic (ROC) curves were then generated by using predictive probabilities to evaluate the diagnostic value. The threshold with the maximum Youden index was chosen as the best threshold. The sensitivity, specificity, and accuracy of the quantitative parameters and qualitative analyses were compared using the McNemar test. The 2-year results of the cumulative early recurrence rate, and the overall survival rate were estimated using the Kaplan-Meier statistical method. The overall survival rate was calculated from diagnosis. α = 0.05 was defined as the level of significance, and *P* > α suggested no statistically significant difference.

## References

[1] Curado MP, Hashibe M (2009). Recent changes in the epidemiology of head and neck cancer. Curr Opin Oncol..

[2] Rzewnicki I, Biszewska J (2013). Epidemiology of laryngeal and hypopharyngeal cancer in the period 1988–2012 in the material of the Otolaryngology Clinic of the Bialystok Medical University. Otolaryngologia polska. The Polish otolaryngology.

[3] Grã©Goire V, Lefebvre JL, Licitra L, Felip E (2010). Squamous cell carcinoma of the head and neck: EHNS-ESMO-ESTRO Clinical Practice Guidelines for diagnosis, treatment and follow-up. Annals of Oncology.

[4] Jang JY (2016). Comparison of Oncological and Functional Outcomes between Initial Surgical versus Non-Surgical Treatments for Hypopharyngeal Cancer. Annals of surgical oncology.

[5] David, J. M. *et al*. Treatment at high‐volume facilities and academic centers is independently associated with improved survival in patients with locally advanced head and neck cancer. *Cancer* **123** (2017). 10.1002/cncr.30843 28640546

[6] Nwizu, T., Ghi, M. G., Cohen, E. E. & Paccagnella, A. The role of chemotherapy in locally advanced head and neck squamous cell carcinoma. *Semin Radiat Oncol*. **22**, 198–206. 10.1016/j.semradonc.2012.03.004 22687944

[7] Induction chemotherapy plus radiation compared with surgery plus radiation in patients with advanced laryngeal cancer. The Department of Veterans Affairs Laryngeal Cancer Study Group. *The New England journal of medicine* **324**, 1685–1690 (1991). 10.1056/NEJM199106133242402 2034244

[8] Forastiere AA (2003). Concurrent chemotherapy and radiotherapy for organ preservation in advanced laryngeal cancer. The New England journal of medicine.

[9] Bonner JA (2006). Radiotherapy plus cetuximab for squamous-cell carcinoma of the head and neck. The New England journal of medicine.

[10] Silver CE, Beitler JJ, Shaha AR, Rinaldo A, Ferlito A (2009). Current trends in initial management of laryngeal cancer: the declining use of open surgery. Eur Arch Otorhinolaryngol..

[11] Marur S, Forastiere AA (2016). Head and Neck Squamous Cell Carcinoma: Update on Epidemiology, Diagnosis, and Treatment. Mayo Clin Proc..

[12] Machiels, J. P. *et al*. Advances in the management of squamous cell carcinoma of the head and neck. *1000Prime* **6**(44), 10.12703/P12706-12744. eCollection 12014 (2014). 10.12703/P6-44 PMC4047945 24991421

[13] Vogl TJ (2012). Dual-energy CT applications in head and neck imaging. AJR Am J Roentgenol..

[14] Fleischmann D, Boas FE (2011). Computed tomography–old ideas and new technology. Eur Radiol..

[15] Ko JP, Brandman S, Stember J, Naidich DP (2012). Dual-energy computed tomography: concepts, performance, and thoracic applications. J Thorac Imaging..

[16] van Elmpt W, Landry G, Das M, Verhaegen F (2016). Dual energy CT in radiotherapy: Current applications and future outlook. Radiotherapy and oncology: journal of the European Society for Therapeutic Radiology and Oncology.

[17] Chen A (2016). Application of dual-energy spectral CT imaging in differential diagnosis of bladder cancer and benign prostate hyperplasia. Medicine.

[18] Yang L (2016). Differentiation of malignant cervical lymphadenopathy by dual-energy CT: a preliminary analysis. Scientific reports.

[19] Zhao Y (2017). Preliminary study on the diagnostic value of single-source dual-energy CT in diagnosing cervical lymph node metastasis of thyroid carcinoma. Journal of thoracic disease.

[20] Mak MP, Glisson BS (2014). Is there still a role for induction chemotherapy in locally advanced head and neck cancer?. Curr Opin Oncol..

[21] Loo SW, Geropantas K, Roques TW (2013). Functional organ preservation in locally advanced laryngeal squamous cell carcinoma: is there a role for induction chemotherapy?. Clin Oncol (R Coll Radiol)..

[22] Rezvani M, Fowler JF, Hopewell JW, Alcock CJ (1993). Sensitivity of human squamous cell carcinoma of the larynx to fractionated radiotherapy. The British journal of radiology.

[23] Wu W (2009). Hemoglobin-based oxygen carriers combined with anticancer drugs may enhance sensitivity of radiotherapy and chemotherapy to solid tumors. Artificial cells, blood substitutes, and immobilization biotechnology.

[24] Dhani N, Fyles A, Hedley D, Milosevic M (2015). The clinical significance of hypoxia in human cancers. Semin Nucl Med..

[25] Hill RP (2015). Hypoxia and Predicting Radiation Response. Semin Radiat Oncol..

[26] Boll DT (2010). Focal cystic high-attenuation lesions: characterization in renal phantom by using photon-counting spectral CT–improved differentiation of lesion composition. Radiology.

[27] Fletcher JG (2009). Dual-energy and dual-source CT: is there a role in the abdomen and pelvis?. Radiologic clinics of North America.

[28] Goodsitt MM, Christodoulou EG, Larson SC (2011). Accuracies of the synthesized monochromatic CT numbers and effective atomic numbers obtained with a rapid kVp switching dual energy CT scanner. Medical physics.

[29] Landry G (2011). Extracting atomic numbers and electron densities from a dual source dual energy CT scanner: experiments and a simulation model. Radiotherapy and oncology: journal of the European Society for Therapeutic Radiology and Oncology.

[30] Yamauchi H, Buehler M, Goodsitt MM, Keshavarzi N, Srinivasan A (2016). Dual-Energy CT-Based Differentiation of Benign Posttreatment Changes From Primary or Recurrent Malignancy of the Head and Neck: Comparison of Spectral Hounsfield Units at 40 and 70 keV and Iodine Concentration. AJR. American journal of roentgenology.

[31] Li JH, Du YM, Huang HM (2015). Accuracy of dual-energy computed tomography for the quantification of iodine in a soft tissue-mimicking phantom. J Appl Clin Med Phys..

[32] Wang L (2012). Correlation between CT attenuation value and iodine concentration *in vitro*: discrepancy between gemstone spectral imaging on single-source dual-energy CT and traditional polychromatic X-ray imaging. J Med Imaging Radiat Oncol..

[33] Li GJ (2016). Correlation between vascular endothelial growth factor and quantitative dual-energy spectral CT in non-small-cell lung cancer. Clinical radiology.

[34] Hellbach K (2017). Dual energy CT allows for improved characterization of response to antiangiogenic treatment in patients with metastatic renal cell cancer. European radiology.

[35] Kumar R, Indrayan A (2011). Receiver operating characteristic (ROC) curve for medical researchers. Indian pediatrics.

[36] Metz CE (1978). Basic principles of ROC analysis. Semin Nucl Med..

[37] Cohen J (2015). Basic principles of diagnostic evaluation. Arch Pediatr..

[38] Forastiere AA (2003). Concurrent chemotherapy and radiotherapy for organ preservation in advanced laryngeal cancer. N Engl J Med..

[39] Schaefer U, Micke O, Schueller P, Willich N (2000). Recurrent head and neck cancer: retreatment of previously irradiated areas with combined chemotherapy and radiation therapy-results of a prospective study. Radiology..

[40] Gleich LL (2004). Recurrent advanced (T3 or T4) head and neck squamous cell carcinoma: is salvage possible?. Arch Otolaryngol Head Neck Surg..

[41] Ganly I, Kaye SB (2000). Recurrent squamous-cell carcinoma of the head and neck: overview of current therapy and future prospects. Annals of oncology: official journal of the European Society for Medical Oncology/ESMO.

[42] Pinto HA, Jacobs C (1991). Chemotherapy for recurrent and metastatic head and neck cancer. Hematol Oncol Clin North Am..

[43] Edge SB, Compton CC (2010). The American Joint Committee on Cancer: the7th edition of the AJCC cancer staging manual and the future of TNM. Ann Surg Oncol..

